# Editorial: Genetic regulation of reproduction traits in livestock species

**DOI:** 10.3389/fgene.2024.1481463

**Published:** 2024-09-05

**Authors:** M. M. D. Barbero, S. Lyu, F. Cendron, N. Song

**Affiliations:** ^1^ Departmento de Genética, Instituto de Ciências Biológicas e da Saúde, Universidade Federal Rural do Rio de Janeiro, Rio de Janeiro, Brazil; ^2^ The Shennong Laboratory, Institute of Animal Husbandry, Henan Academy of Agricultural Sciences, Zhengzhou, China; ^3^ Department of Agronomy, Food, Natural Resources, Animals and Environment, University of Padova, Viale dell’Università, Legnaro, Italy; ^4^ College of Animal Science and Technology, Anhui Agricultural University, Hefei, China

**Keywords:** candidate genes, CNV (copy number variation), fertility, SNP, omics

The genetic regulation of reproduction traits in livestock species is an important area of study within animal science and genetics. Understanding the underlying genetic mechanisms that control reproductive traits is essential for improving breeding programs and enhancing productivity. These traits are typically complex, influenced by numerous genes, few of which have predominant effects, and are also influenced by environmental factors. Examples of reproduction traits include fertility, litter size, gestation length, and puberty onset. Advances in molecular biology and multi-omics have provided powerful tools to identify the genetic factors that affect reproduction traits. Studies in this subject aim not only to improve the efficiency and sustainability of livestock production but also contribute to animal welfare and the economic viability of the livestock industry. This Research Topic encompasses seven articles highlighting different Research Topic currently being studied, summarized below, offering valuable insights into this field.

Jia et al., studied the egg-laying in a Chinese local breed, Wuhua yellow-feathered chicken. This trait is strongly associated with chicken follicle development, which is controlled by the gonadal axis of the reproductive endocrine system. During this process RNA modifications, such as in RNA N6-methyladenosise (m6A), occur. These authors conducted a transcriptome-wide m6A methylation analysis of Wuhua yellow-feathered chicken ovaries before and after sexual maturation to identify the potential molecular mechanisms underlying chicken ovary development. A positive correlation was observed between the m6A peaks and gene expression levels, indicating that m6A may play an important role in the regulation of chicken ovary development. Functional enrichment analysis indicated that apoptosis-related pathways could be the key pathway underlying the poor reproductive performance of Wuhua yellow-feathered chicken.

Follicular atresia is the process through which ovarian follicles degenerate and are reabsorbed before reaching maturity. This is a natural part of the ovarian cycle and occurs throughout a female’s reproductive life. Studies have shown that miRNAs influence the follicular atresia by post-transcription regulation. Liu et al., performed a high-throughput small RNA sequencing to analyze differential miRNA expression profiles between healthy (HF) follicles and early atretic (EAF) follicles. A total of 237 conserved miRNAs were detected, and miR-143 is the highest expressed in follicles. Additionally, 22 differentially expressed miRNAs in EAF compared to HF were identified. Enrichment analysis for the target genes of differentially expressed miRNAs showed these miRNAs contribute to follicular atresia initiation and cell fate.

Follicle development depends on various factors, including GCs growth and hormone production. Long noncoding RNAs (LncRNAs) are known to play essential regulatory roles in follicle development. In a previous study, Wang et al., observed differential expression of *Loc105611671* in Qira black sheep during the pre-estrus and estrus phases. Now analyzed into the influence of a Loc105611671, on the proliferation and steroid hormone synthesis of sheep ovarian GCs and the associated target genes *in vitro*. Overexpression of *Loc105611671* increased the GCs proliferation, along with estrogen (E2) and progesterone (P4) levels, and interacts with CDC42 causing an upregulation of the expression of this protein.

Hess et al. estimated the heritability of pubertal classification using a univariate Bayesian animal model, finding values ranging from moderate to high. A genome-wide association study (GWAS) was performed but did not identify single nucleotide polymorphisms (SNPs) significantly associated with pubertal classifications. Consequently, a candidate gene approach was employed, identifying eight genes/regions associated with pubertal classifications and twenty-two genes/regions associated with whether puberty was attained during the trial. Additionally, whole genome sequencing (WGS) data from 33 heifers were aligned to identify variants in FSHR, a gene critical for pubertal attainment. Fisher’s exact test was used to determine if FSHR SNPs segregated by pubertal classification. Genotyping analysis showed one FSHR SNP was associated with pubertal classifications and cyclicity during the trial.

Copy number variation (CNV) refers to structural variations in the genome where the number of copies of a particular gene or genomic region varies between individuals. These variations can occur anywhere in the genome and can influence gene expression and phenotypic traits. Zhang et al. examined the CNVs in Wanbei pigs (WBP) and Asian wild boars (AWB) using whole genome resequencing data. They identified that 1.20% and 1.33% of the genome contain CNVs in WBP and AWB, respectively. Based on the top 1% of fixation index values, 164 CNVs under selection were determined. Functional enrichment analyses of the genes associated with these CNVs revealed links to reproduction (SPATA6, CFAP43, CFTR, BPTF), growth and development (NR6A1, SMYD3, VIPR2), and immunity (PARD3, FYB2).

Zhao et al. performed a whole-genome resequencing of local Chinese dairy goat breeds and imported breeds., and their differences in selection signals, genomic genetic variation, genetic diversity, and population structure were subsequently identified. Candidate genes related some important traits of dairy goats corresponding to selection signals were explored, and significant findings included: milk production (*STK3*, *GHR*, *PRELID3B*), reproduction (*ATP5E*), growth and development (*CTSZ*, *GHR*), and immune function (*CTSZ*, *NELFCD*). Furthermore, allele frequency distributions for these genes between the two populations were revealing significant differences.

The last article accepted, Gonzalez-Berrios et al. identified and validated SNP associated with fertility traits that impact in the early embryo mortality from mRNA sequencing information. Validation of candidate SNP and genotype to phenotype analysis were conducted in a separate cohort of lactating primiparous Holstein cows. Significant associations with fertility traits were found in SNPs within the genes DSC2, SREBF1, UBD, DECR1, FASN, SREBF1 and BOLA-DMB. Only DSC2 SNP had an important allele substitution effect in cows with the G allele by having a decreased age at first calving by 10 days.

The findings in some of these articles can be utilized to develop genetic selection tools such as marker-assisted selection or by incorporating the identified SNPs into a SNP panel. Furthermore, the study offers a valuable resource for investigating genetic diversity and the conservation of native breeds. These articles also highlight candidate genes that can be sequenced to identify causal mutations or as targets for gene editing. In addition to their practical applications, these studies provide some new insights into the molecular mechanisms underlying reproductive traits.

